# Early development of vocal interaction rules in a duetting songbird

**DOI:** 10.1098/rsos.171791

**Published:** 2018-02-21

**Authors:** Karla D. Rivera-Cáceres, Esmeralda Quirós-Guerrero, Marcelo Araya-Salas, Christopher N. Templeton, William A. Searcy

**Affiliations:** 1 Department of Biology, University of Miami, Coral Gables, FL 33146, USA; 2 School of Biology, University of St Andrews, St Andrews, UK; 3 Laboratory of Ornithology, Cornell University, Ithaca, NY, USA; 4 Laboratorio de Bioacústica, Escuela de Biología, Universidad de Costa Rica, San Pedro, San José, Costa Rica; 5 Department of Biology, Pacific University, Forest Grove, OR 97166, USA

**Keywords:** vocal interaction, duetting, duet codes, duet development, temporal coordination, birdsong

## Abstract

Exchange of vocal signals is an important aspect of animal communication. Although birdsong is the premier model for understanding vocal development, the development of vocal interaction rules in birds and possible parallels to humans have been little studied. Many tropical songbirds engage in complex vocal interactions in the form of duets between mated pairs. In some species, duets show precise temporal coordination and follow rules (duet codes) governing which song type one bird uses to reply to each of the song types of its mate. We determined whether these duetting rules are acquired during early development in canebrake wrens. Results show that juveniles acquire a duet code by singing with a mated pair of adults and that juveniles gradually increase their fidelity to the code over time. Additionally, we found that juveniles exhibit poorer temporal coordination than adults and improve their coordination as time progresses. Human turn-taking, an analogous rule to temporal coordination, is learned during early development. We report that the ontogeny of vocal interaction rules in songbirds is analogous to that of human conversation rules.

## 1. Introduction

Complex communication systems, including human language, involve highly structured interactions [1]. Vocal interactions between other types of primates include call exchanges between group members in marmoset monkeys [2] and duetting between mated pairs of gibbons [3]. Among non-human species, however, the most complex vocal interactions studied to date occur in birds [4]. Male songbirds engage in various kinds of vocal interactions during aggressive encounters such as counter-singing [5,6], song type matching [7], repertoire matching [8] and frequency matching [9]. In all of these cases, escalation in aggressiveness depends on the type of responses individuals give to each other [10]. Perhaps the most complex vocal interaction among birds is duetting between mated males and females, a behaviour that is particularly common in tropical species [11].

To be able to engage in any of these vocal interactions, individuals must develop not only the ability to produce their vocalizations but also the ability to follow the correct conventions in using those vocalizations in replying to others. Previously, studies of the development of acoustic vocal signals have concentrated on the development of the structure of individual vocalizations [12–14]. These studies have shown strong analogies between the development of human speech and birdsong [13]. For instance, vocal production learning (i.e. the ability to modify the structure of vocalizations as a consequence of hearing others) occurs during early development in birds and humans but not in non-human primates [15] (but see [16] for an exception). However, it is unknown if analogies also exist between the development of vocal interaction rules in humans and birds.

A few studies have addressed the development of vocal interaction rules in non-human primates, finding some analogies with human interaction rules. For instance, recent work suggests that turn-taking, the ability to exchange utterances rapidly but without overlap [17], is learned during early development in humans [18]. This ability seems to be learned also in marmosets (*Callithrix jacchus*) [19]. It has been also suggested that in agile gibbons (*Hylobates agilis agilis*), mother–daughter interactions enhance vocal development and allow juveniles to learn temporal patterns needed to engage in duet singing [20]. In humans, interactions between carers and infants enhance speech learning [21]. These results suggest that both, species that learn to produce their vocalizations and those that do not (as non-human primates [15,22]) can learn vocal interaction rules, and thus that vocal production learning and vocal interaction learning do not need to be tied (but see [16,22] for evidence of vocal production learning in marmosets). However, vocal production learning has led to vocal repertoires becoming more complex [12], thus providing an opportunity for the evolution of more complex vocal interaction rules such as those found in human conversation. Thus, it is key to determine whether songbirds (the most thoroughly studied vocal production learners) possess the ability to learn vocal interaction rules (i.e. ‘vocal interaction learning hypothesis’).

Two studies have indirectly addressed the early ontogeny of vocal interactions in temperate avian species. First, it has been shown that common nightingales (*Luscinia megarhynchos*) during early development learn not only individual song types, but also the order in which a group of songs is delivered [23]. Second, when juvenile nightingales are exposed to a series of groups of songs in a particular order, they also distinguish the sequential association of the different song groups [24]. It has been argued that these two features suggest that juveniles learn contextual information of when and how the songs should be used during vocal interactions [4]. However, these tests were carried out without the occurrence of vocal interactions between individuals and thus only reflect the individual's ability to learn appropriate song sequences. Thus, no direct test of the vocal interaction learning hypothesis has been performed in avian species.

Duetting species must engage in vocal interactions that involve time and pattern-specific relationships among the vocalizations from different individuals. In one duetting species, the canebrake wren (*Cantorchilus zeledoni*), individuals can acquire new interaction rules in adulthood when they obtain new mates, and the new rules develop gradually, which is suggestive of learning [25]. In black-bellied wrens (*Pheugopedius fasciatoventris*), adults are able to answer to unfamiliar songs, suggesting that they can learn new rules to answer these songs [26]. Furthermore, juveniles of some duetting species duet with adults during the sub-song stage (e.g. buff-breasted wrens, *Cantorchilus leucotis* [27], black-bellied wrens [28] and canebrake wrens, personal observation) in a way similar to the way infant humans converse with their carers [21]. Finally, a hand-rearing experiment in bay wrens (*Cantorchilus nigricapillus*) showed that social interactions are essential for young birds to learn the correct song repertoires [29]. It is then probable that the early ontogeny of these rules in some duetting species also involves learning, and thus these species provide an ideal model to address the vocal interaction learning hypothesis.

Many duetting birds must abide by two interaction rules: non-random association of duet types (i.e. duet codes [26]) and precision in the timing of song answering (analogous to turn-taking in primates [5]). The former rule is absent in non-human primates. However, humans possess an analogous rule termed ‘adjacency pairs' in which exchanges of types of utterances are linked (e.g. question–answer type of utterances) [30]. In this study, we address whether juvenile canebrake wrens gradually acquire the ability to duet and thus acquire both proper coordination and specific duet codes while they sing duets with adults. Regarding duet coordination, the vocal interaction learning hypothesis predicts that juveniles (a) should perform duets with poorer coordination than adults and (b) should improve their coordination with time. Regarding duet codes, this hypothesis predicts that juveniles (c) should use the same code as the adults they sing with, (d) should break the code more often than adults and (e) should break the code less as time progresses. We tested these predictions by recording juvenile canebrake wrens for up to two months in the field and then determining how juvenile duets compared to adult duets and how juvenile duets changed over time. To our knowledge, we are the first to provide direct evidence for the hypothesis that duetting birds acquire the ability to duet as juveniles.

Canebrake wrens are an ideal species to study the development of interaction rules because juveniles can be recorded singing with adults and because of the complexity of adult duets in this species. Adult pairs of canebrake wrens sing highly coordinated antiphonal (i.e. alternating) duets and associate their song types non-randomly (i.e. possess duet codes [31]). Duets in this species are composed of three categories of phrases. Two of these categories are sung by males: introductory phrases (I phrases), which are used to begin songs, and a separate set of male phrases (M phrases), which are used later in the song. The remaining category is the set of phrases sung by the females (F phrases). Duets typically start with a single I phrase followed by an alternation of F and M phrases (i.e. I(FM)_n_). When a juvenile is present on a territory, it usually joins the duets of the adults by singing phrases specific to one sex.

## 2. Methods

In the months of April–August of 2012–2016, we recorded duets performed by groups of territorial plain wrens composed of an adult female, an adult male and at least one juvenile. We started recording juveniles as soon as they were found; however, we were not able to determine the exact age of the juveniles as this species has little reproductive synchronization and we did not band nestlings in the nest [32]. Most individuals from the territories that we recorded were captured and provided with a unique combination of coloured leg bands for further identification. In total, we recorded eight groups (16 adults and 13 juveniles) around La Suerte Biological Station, Costa Rica (10°26′ N, 83°47′ W) and La Selva Biological Station, Costa Rica (10°26' N, 83°59′ W). The study sites include a mixture of lowland moist forest, swamps, scrub and cattle pasture, where canebrake wrens are common [31].

To confirm the genetic sex of the juveniles, we obtained blood samples (approx. 50 ml) from the brachial vein and stored them in lysis buffer [33] for nine out of the 13 juveniles. We extracted the DNA using DNeasy Blood & Tissue Kits (Qiagen). DNA concentrations were measured using a Qubit 2.0 fluorometer (Life Technologies). We ran PCR amplification using the P0, P2 and P8 primers [34]. PCRs were successful for seven out of the nine individuals as we were able to obtain clear bands in agarose gel identifying them as males or females.

### 2.1. Data collection

Territorial birds were recorded between 6.00 and 9.00. To obtain recordings of duets, we used a Marantz PMD660 digital recorder and a unidirectional Sennheiser ME66 microphone (recording format PCM-16, sample rate 44.1 k at 16-bit quantization). Recordings were made on each territory at least once a week for 1 h (average of days recorded per juvenile: 24.7 ± 17.2 s.d.). The recording session started with a simulated intrusion using playback to increase the singing activity of the focal birds. The playback consisted of three bouts of canebrake wren duet songs. The playback was then repeated every 10 min until the recording session was over.

During each recording session two observers were present. One observer performed the recordings and the other observer followed the banded individuals with binoculars to determine which individuals sang each time. Only the duets in which both observers agreed on the individuals that had participated were analysed.

All animal manipulation was approved by the Institutional Animal Care and Use Committee (IACUC) of the University of Miami (Protocol 15–064). This research was performed under a scientific research permit (no. 05354) provided by the Ministry of Environment, Energy and Telecommunications (MINAET) of Costa Rica.

### 2.2. Data analysis

To analyse all duets, we created spectrograms of each recording using the SYRINX software (J. M. Burt) with a Hanning window and a 512 pt FFT and a temporal resolution of 5.8 ms. All duets in which at least one adult and one juvenile sang were analysed. For territories in which both female and male juveniles were present we also included the duets performed solely by the juveniles.

To measure duet coordination, we determined whether each bird overlapped a song of a bird from the opposite sex by subtracting the end time of a phrase from the start time of the next immediate phrase from a bird of the opposite sex. We counted a phrase as overlapped if the result of the subtraction was negative. We then calculated the proportion of phrases overlapped per duet in R (v. 2.15.1) using the function coor.sing from the warbleR package [35].

To classify the phrase types used by each bird in every duet, we created a library of each bird's repertoire. We then compared the classified phrase types to each duet spectrogram. If an adult and a juvenile of the same sex both sang during a duet, we determined whether the phrase types that they used were the same or not. The phrase types were determined based on visual inspection [36].

### 2.3. Statistical analysis

To determine whether juveniles performed less coordinated duets than adults, we used a generalized mixed model (GLMM, function lme of the package nlme [37] in R v. 2.15.1). We used age of bird (adult or juvenile) as a fixed factor and sex and year as covariates (electronic supplementary material, table S1). Because year and sex were non-significant (*p* = 0.35 and *p* = 0.28, respectively), they were removed from the model. We performed a second generalized mixed model to determine whether juveniles improved their coordination with time (electronic supplementary material, table S1). We used day as a fixed factor and sex and year as covariates. Sex and year were dropped from the final model (sex *p* = 0.30, year *p* = 0.31).

To determine if juveniles broke the code more often than adults, we used a third GLMM (electronic supplementary material, table S1). We used age of birds (juvenile versus adult) as a fixed factor. Sex and year were used as covariates, but were dropped from the final model (sex *p* = 0.40, year *p* = 0.28). A fourth GLMM was used to determine whether juveniles improved their duet code adherence with time. Day since first recording and type of duet (duet with both adults, duet with adult of the opposite sex, duet between juveniles) were used as fixed factors. Sex and year were used as covariates (electronic supplementary material, table S1). Sex was dropped from the model (*p* = 0.57) but year was retained. Juveniles sang significantly more phrase types in 2013 than in 2014 (effect size = 1.13, *t*_6_ = 4.41, *p* = 0.004) and 2016 (effect size = 0.85, *t*_6_ = 3.43, *p* = 0.01).

To determine if juveniles broke the code more when they only sang with the adult of the opposite sex, we performed a fifth GLMM (electronic supplementary material, table S1) where presence or absence of adult of the opposite sex was used as fixed factor and sex and year were used as covariates. Both covariates were dropped from the final model (sex *p* = 0.6, year *p* = 0.75).

For all GLMMs we used identity of the bird as a random factor as multiple duets from each individual were used in the analyses. Furthermore, we used territory as a second random factor to control for group effects (electronic supplementary material, table S1). All GLMMs were validated using the graphic methods suggested by Zuur *et al*. [38].

To determine if juveniles used the same code as adults, we compared the song types used by juveniles and the adults of the same sex to answer each song type of the adult of the opposite sex. A heterogeneity G-test was applied to determine if the phrases that juveniles used to answer were chosen randomly or followed the same code as the adults. To calculate the expected values for the contingency table, we used the inverse of the total repertoire size recorded from each juvenile.

## 3. Results

### 3.1. Sex-specific repertoires

Each of the 13 juveniles we recorded sang either only male phrases or only female phrases. We determined the genetic sex for seven juveniles: three females and four males. In all seven cases, the juveniles sang phrases that were appropriate for their genetic sex—that is, males sang male phrases and females sang female phrases.

### 3.2. Duet coordination

**Figure 1.**
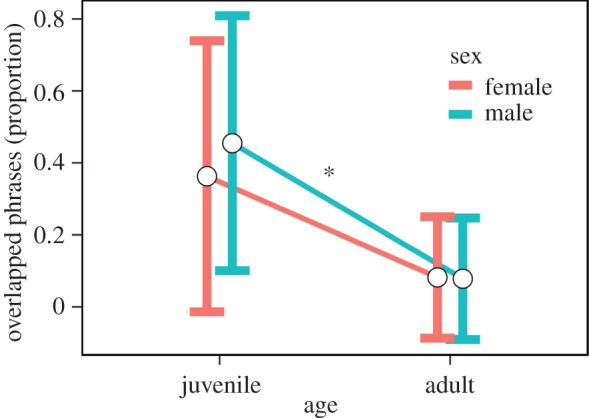
Proportion of phrases of the opposite sex per duet that females (red) and males (blue) overlapped with their own phrases by age (adults versus juveniles). Average (white circles) and standard deviation (top and bottom vertical whiskers) are shown. *p < 0.01.

**Figure 2.**
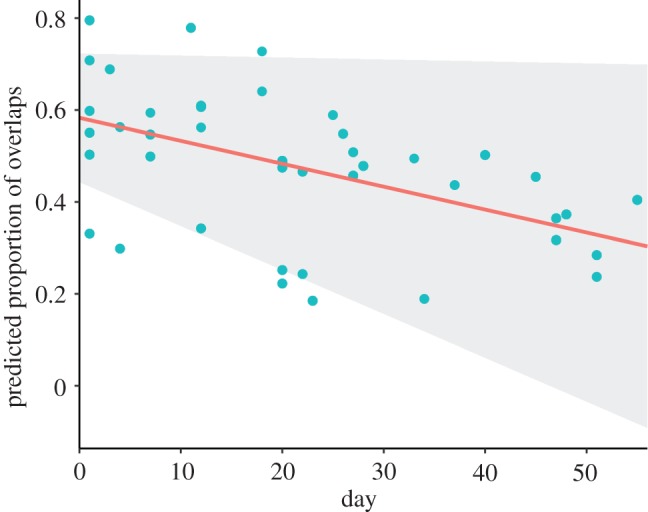
Proportion of phrases of the opposite sex per duet that juveniles overlapped with their own phrases across time. The red line represents the fixed effect of day over the proportion of phrases overlapped. The blue dots represent the predicted values by the model. The shaded area represents the 95% confidence intervals of the GLMM.

**Figure 3.**
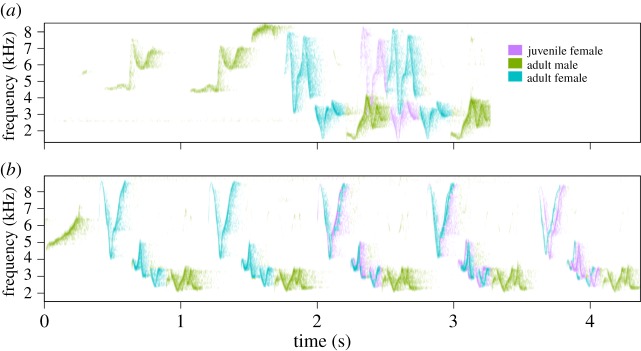
Examples of duets in which female juvenile L2 (magenta) sings with both adults: (*a*) lower coordination during the first day of recording and (*b*) higher coordination two weeks after the first day of recording. Adult female is shown in blue and adult male is shown in green.

Juvenile canebrake wrens overlapped significantly more songs of the opposite sex than did adults and thus exhibited poorer coordination (effect size = 0.425, *t*_24_ = 7.75, *p* < 0.00001, figure 1). Furthermore, juveniles significantly improved their coordination over time within the time frame measured (average days recorded per juvenile 24.69 ± 17.2 s.d.). Although the effect size per day was small, overall juveniles decreased their overlapping proportion by about 20% over the total time each was recorded (effect size = −0.004, *t*_315_ = −2.57, *p* = 0.01, figures 2 and 3). However, there is considerable variation in the rate at which juveniles decreased duet overlapping (figure 2).

### 3.3. Duet code adherence

**Figure 4.**
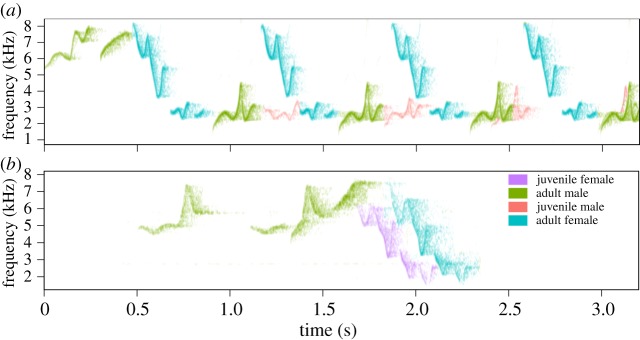
Examples of duets in which juveniles use the same duet code as adults. (*a*) An ‘F' phrase of an adult female (blue) is answered by the adult (green) and juvenile (red) males; both males used the same phrase type. (*b*) An ‘I' male phrase (green) is answered by the adult (blue) and juvenile (purple) females; both females used the same phrase type.

**Figure 5.**
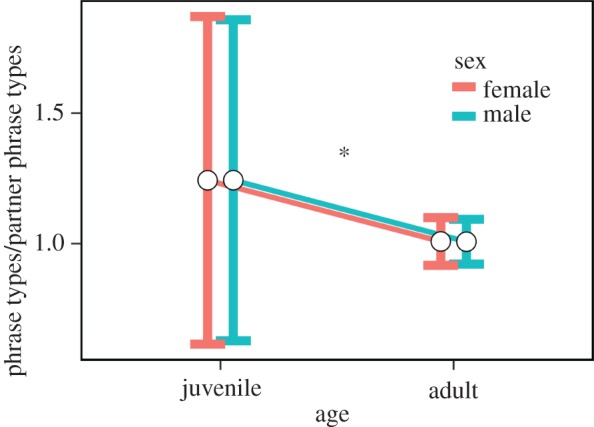
Number of phrase types that females (red) and males (blue) used to answer each phrase type of the adult of the opposite sex by age. Average (white circles) and standard deviation (top and bottom vertical whiskers) are shown. *p < 0.01.

All 13 juveniles matched the phrase type that the adult of the same sex used to answer phrases from the opposite sex with a probability far above chance (*G*_tot_ = 1490.2, d.f. = 13, *p* < 0.0001, table 1, figure 4). These results indicate that juveniles use the same duet code as the adults they duet with. Between individuals, however, there was significant heterogeneity in terms of how strictly they followed the adult duet code (*G*_het_ = 65.67, d.f. = 12, *p* < 0.0001). Additionally, juvenile canebrake wrens used more phrase types to answer the adult from the opposite sex than did adults (effect size = 0.283, *t*_24_ = 3.26 *p* = 0.0032, figure 5). That is, juveniles usually answered with the same song type as adults from the same sex, but not exclusively using that one song type within a duet. This result shows that the duet codes of juveniles are less consistent than the duet codes of adults.

**Table 1.** G tests for whether juveniles matched the duet codes of the parents by answering with the same phrase type as their same-sex parent to each of the phrase types of their opposite-sex parent. M = phrases from juveniles that matched the same-sex adult phrase type, NM = phrases from juveniles that did not match the same-sex adult phrase type, EM = expected matches, ENM = expected non-matches.

individual	M	NM	total	EM	ENM	*G*	d.f.	*p*
G2F 2013	21	9	30	3.75	26.25	53.088	1	<0.0001
G2M 2013	18	17	35	4.375	30.625	30.908	1	<0.0001
H1M 2012	25	3	28	3.5	24.5	85.705	1	<0.0001
L2F 2013	52	1	53	6.625	46.375	206.61	1	<0.0001
PAEBF 2013	17	4	21	2.625	18.375	51.319	1	<0.0001
PAEBM 2013	24	2	26	3.25	22.75	86.245	1	<0.0001
G2F 2016	50	2	52	6.5	45.5	191.52	1	<0.0001
G2M 2016	95	3	98	12.25	85.75	369.07	1	<0.0001
G4 F 2015	42	5	47	5.875	41.125	144.15	1	<0.0001
G4 M 2015	24	3	27	3.375	23.625	81.777	1	<0.0001
BG1 M 2015	41	7	48	6	42	132.5	1	<0.0001
LS2 F 2016	18	6	24	3	21	49.47	1	<0.0001
LS2 M 2016	4	3	7	0.875	6.125	7.876	1	<0.005
					*G*_total_	1490.2	13	<0.0001
					*G*_pooled_	1424.6	1	<0.0001
					*G*_heterogeneity_	65.678	12	<0.0001

**Figure 6.**
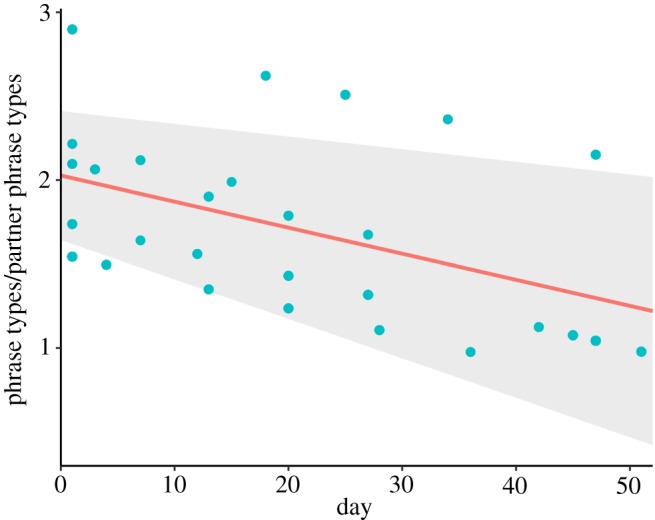
Number of phrase types that juveniles used to answer each phrase type of the adult of the opposite sex across time. The red line represents the fixed effect of day over the number of phrase types used. The blue dots represent the predicted values by the model. The shaded area represents the 95% confidence intervals of the GLMM.

Furthermore, juveniles used more phrase types when they duetted only with the adult of the opposite sex (1.87 ± 1.08 average ± s.d.) than when they duetted with both adults (1.17 ± 0.53 average ± s.d.) (effect size = −0.83, *t*_317_ = −6.50, *p* < 0.00001). These results suggest that juveniles use the song of the same-sex adult as a template to copy the correct response in real time to be able to answer the adult of the opposite sex. Finally, juveniles used fewer phrase types to answer one phrase type of the opposite sex as time progressed (effect size = −0.015, *t*_318_ = −3.83, *p* < 0.0001, figure 6), which indicates that their use of a defined duet code improved with time.

## 4. Discussion

### 4.1. Duet code development

This is the first study that provides direct evidence that a duetting bird gradually acquires a duet code during early development. We argue that this acquisition is the result of learning from duetting with adults rather than simple physiological or motivational maturation of the juvenile. First, we showed that juvenile canebrake wrens use the same code as the adults they duet with, adhering to duet codes that differ between family groups [25,39]. Adherence to a particular duet code means that each juvenile selects a specific song from its learned repertoire to answer each of the opposite-sex adult's songs. Consistent choice of the same songs that the adult model uses could only be accomplished by copying, not by maturation. Second, juveniles used the correct duet code less often when the adult from the same sex was not singing in the duet (i.e. when the juvenile was duetting only with the adult of the opposite sex). This result suggests that the song choices of the same-sex adult directly prompt the choices made by the juvenile. Third, juveniles improve with time in their duet code adherence, suggesting that with practice juveniles are able to store the correct duet code in their memories.

A recent study showed that the duet codes of adult canebrake wrens change when they acquire a new partner and are thus flexible [25]. A second duetting species that is thought to have flexible duet codes is the black-bellied wren [40], as individuals are able to answer to unfamiliar phrases. The question then remains of why juveniles should learn a duet code from their parents if they have to invest in relearning new rules when they mate. One possibility is that what is important for juveniles to learn may be the general rules governing duet codes (i.e. knowing they have to pair specific songs in their own repertoire with specific songs in their partners' repertoires), rather than the specifics of the codes used by their models. A hand-rearing experiment depriving juveniles of early exposure to duet codes could be used to test this idea.

This proposed learning pattern is similar to human conversation learning. In humans, higher cognitive skills needed to exchange ideas (such as the social manifestation of differences in perspective) can be learned throughout life, but exposure to speech interactions during early development is nevertheless vital for the general ability of individuals to engage in conversations [41]. Gibbons may also share this pattern as there is socially mediated vocal development in early stages with some flexibility in duetting performance in sub-adult and adult stages [20].

The study of the development of adjacency pairs, the human rule that is analogous to duet codes in birds, has been difficult. The adjacency pairs rule can encompass many scenarios within conversations as the general idea is that the rule is fulfilled if an individual makes a conversational contribution as is required, at the correct stage with the accepted purpose [42]. Thus, protocols that can objectively target measurements of rule adherence can be difficult to generate. Still, by reviewing conversations of children around 2 years of age, researchers in conversations analysis have been able to determine that these young children can coherently relate to what was said by the previous speaker and frame their response accordingly. Thus, it seems that children develop the ability to engage in the collaborative activity that is required for adjacency pairs early in life [43]. However, it is still unknown what the connectors between exchanges are and how explicit they have to be so that children understand their role in the conversation at this age. Furthermore, it is unknown how and at what age this competence starts to develop. In this study, we have found that duet codes develop during early development in a similar manner to adjacency pairs. It seems that juvenile canebrake wrens require both adults to duet with them to start acquiring the connectors between I-F-M phrases (i.e. juveniles tend to make fewer mistakes when the parent of the same sex sings), but as time progresses, juveniles are potentially memorizing the connectors and can duet with the parent of the opposite sex without the need of input from the same-sex parent. It is possible that children also need to listen to third-party conversations to understand the general rule of adjacency pairs. Perhaps comparative analyses of children growing up in different conditions (e.g. foster homes versus family homes) and their ability to find connectors in a cooperative conversation could help in the understanding of the mechanism of how this rule is acquired. Experimental studies with juvenile wrens could also help us understand whether duetting birds indeed need to hear duetting from other individuals to be able to develop their duet code rules.

### 4.2. Duet coordination development

Both the lower coordination performance of juvenile canebrake wrens and their coordination improvement through time support the idea that juveniles need a rehearsal period to be able to coordinate their duets. Thus, here we provide evidence that an analogous rule to turn-taking, i.e. temporal coordination in duetting birds, also has an analogous development. In both humans and canebrake wrens, this ability is acquired during early development. To our knowledge, this is the first study to show that birds and humans not only share the ontogeny of vocalizations acquisition but also the ontogeny of the rules needed to use those vocalizations during vocal interactions.

A recent study in marmoset monkeys showed that this species also acquires the ability to exchange vocalizations without overlapping during early development, making marmosets the first species other than humans reported to do so [17,19]. However, turn-taking in marmosets is somewhat different than in humans because the time frame in which each species performs exchanges differs by several orders of magnitude: the silent gap between the utterances of two humans takes a few hundred milliseconds (mode approx. 200 ms) [44], while silent gaps between calls in marmosets can take up to 10 s (median 5.63 s) [2]. Contrastingly, the silent gap between the phrases of canebrake wrens also happens within less than 100 ms (average females = 0.64 ms males = 0.46 ms) [45]. Furthermore, it has been shown that in order to keep silent gaps short, several species of duetting birds perform adjustments to their singing tempo based on which phrases are sung by their partners [45–48]. Furthermore, as in humans [44], birds in at least one duetting species are able to predict the end of their partners’ vocalization [48]. Although marmosets and humans are fairly closely related, it has been suggested that turn-taking in the two species is analogous and not homologous [19]. For all the above reasons, it may be more informative to compare turn-taking in birds and humans than in non-human primates and humans. Moreover, the birdsong model provides us with the capacity to perform neurological and genetic studies in species that possess many homologous and analogous brain regions and genetic components dedicated to song learning and song production that in humans relate to speech learning and speech production [49].

Our study has provided key evidence that vocal interaction rules are learned during early development, but there is still need of studies that provide further tests of this idea. For instance, in our study the rate of improvement in duet coordination was not consistent throughout all juveniles (i.e. large confidence intervals, figure 2). The lack of a clear pattern of duet coordination improvement could be due to the need for a longer rehearsal period than the time frame of our study. In the field, we only observed one pair of juvenile birds leaving their parental territory, more than two months after beginning to sing. Before they left, they consistently performed highly coordinated duets. It could be that all juveniles achieve consistent high coordination before leaving the natal territory. Other duetting species take up to eight months (in slate-coloured boubous, *Laniarius funebris* [50]) to develop a crystallized song repertoire, and thus it could take the same amount of time to develop the ability to perform highly coordinated duets. To be able to confirm this idea, a longer observation period of juvenile duet development controlling for the stage at which each juvenile was first recorded in the field is warranted.

Furthermore, no studies have directly addressed the development of other types of vocal interactions in birds, but it will be very interesting to determine, for instance, if song type matching in songbirds [5] develops in a similar fashion.

## 5. Conclusion

This study provides evidence that both duet coordination and duet codes are acquired through learning during early development in canebrake wrens: juvenile canebrake wrens improve both their temporal coordination and duet code adherence through time. Furthermore, the duet code that the juveniles acquire is the same as the code of the adults with whom they sing. This is the first study to show that the ontogeny of vocal interaction rules of humans and songbirds are also analogous.

## Supplementary Material

Table S1

## Data accessibility

Datasets are available in Dryad (http://dx.doi.org/10.5061/dryad.k46k2) [51].
