# Supplementary material for: Early development of vocal interaction rules in a duetting songbird
Source: R Soc Open Sci. 2018 Feb 21;5(2):171791. doi: 10.1098/rsos.171791 (PMC5830777; doi:10.1098/rsos.171791)
Supplement: Table S1 [file rsos171791supp1.docx]

Table S1. General Linear Mixed Model structure for all models.

| Model | Description | Dependent variable | Fixed factors | Covariates | Random factors |
| --- | --- | --- | --- | --- | --- |
| M1 | Coordination by age | Proportion of overlap | Age (Juvenile vs. Adults) | Sex*+Year* | Individual+Territory |
| M2 | Coordination through time in juveniles | Proportion of overlap | Day | Sex*+Year* | Individual+Territory |
| M3 | Duet code adherence by age | Number of phrase types individuals used to answer each phrase type of opposite sex. | Age (Juvenile vs. Adults) | Sex*+Year* | Individual+Territory |
| M4 | Duet code adherence through time in juveniles | Number of phrase types juveniles used to answer each phrase type of opposite sex. | Day | Sex*+Year | Individual+Territory |
